# Supplementary material for: Multiple 9-1-1 complexes promote homolog synapsis, DSB repair, and ATR signaling during mammalian meiosis
Source: eLife. 2022 Feb 8;11:e68677. doi: 10.7554/eLife.68677 (PMC8824475; doi:10.7554/eLife.68677)
Supplement: Figure 1—source data 3. — These data were used to create tSNE plots for Figure 1—figure supplement 1B–D. Source code is available at https://github.com/nyuhuyang/scRNAseq-SSCs (copy archived at swh:1:rev:9e17b2b7a8871b9aa4f506fdb723f637fc9f0b2c, Hu, 2022). [file elife-68677-fig1-data3.pdf]

Data to create tSNE plots for figure 1 supplement figures 1B-D can be found at GEO and are accessible through Series accession number GSE121904. Source code is available at: https://github.com/nyuhuyang/scRNAseq-SSCs . Further information can be found at the original paper: https://doi.org/10.1371/journal.pgen.1007810.
